# Ripk2 promotes CD8^+^ T cell inactivation and hepatocellular carcinoma progression through Myb/Cxcl9 and Pax5/Adpgk signaling pathways

**DOI:** 10.1038/s41419-026-08849-0

**Published:** 2026-05-29

**Authors:** Zengbin Wang, Huahui Yu, Le Yu, Mengxin Liu, Xuemei Huang, Linqing Wu, Wenhao Teng, Shiguang Chen, Zhuting Fang

**Affiliations:** 1 Department of Immunology, School of Basic Medical Sciences, Fujian Medical University, Fuzhou, 350122 China; 2 Department of Hepatopancreatobiliary Surgery, Clinical Oncology School of Fujian Medical University, Fujian Cancer Hospital, Fuzhou, China; 3 Department of Oncology and Vascular Interventional Therapy, Clinical Oncology School of Fujian Medical University, Fujian Cancer Hospital, NHC Key Laboratory of Cancer Metabolism, Fuzhou, 350014 China

**Keywords:** Tumour immunology, Tumour heterogeneity, Liver cancer

## Abstract

Hepatocellular carcinoma (HCC) is one of the most common malignancies worldwide, characterized by complex pathogenesis and immune escape mechanisms. The tumor immune microenvironment plays an increasingly recognized role in tumorigenesis, progression, and treatment. Receptor-interacting serine/threonine-protein kinase 2 (Ripk2) plays a pivotal role in inflammatory responses and immune regulation. This study aimed to investigate the role of Ripk2 in the HCC immune microenvironment, particularly its impact on macrophage function, using a macrophage Ripk2 knockout mouse model (Ripk2^*CKO*^), single-cell sequencing analysis (scRNA-seq) and flow cytometry (FCM). We found that inhibiting or deleting Ripk2 altered the intratumoral microbiota within macrophages, significantly increasing the abundance of *Streptomyces collinus* (*Sc*). This alteration not only prompted a metabolic shift in macrophages but also significantly upregulated the transcription factor Myb expression, promoting Cxcl9 secretion, thereby enhancing CD8^+^ T cell infiltration, activity, and proliferation. Furthermore, Ripk2 was found to promote lactate production *via* the Pax5/Adpgk pathway, leading to CD8^+^ T cell dysfunction and forming an immunosuppressive feedback loop in HCC. Combining a Ripk2 inhibitor (GSK583) with a PD-1/PD-L1 inhibitor (BMS202) reduced the hepatotoxicity of BMS202 monotherapy and improved therapeutic efficacy. In conclusion, this study provides new insights into the role of Ripk2 in the HCC immune microenvironment and lays an experimental foundation for the development of Ripk2-targeted immunotherapy strategies.

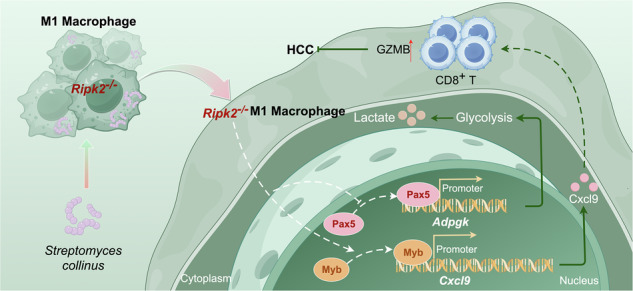

## Introduction

Intratumoral microbiota influence tumor progression through various mechanisms, including interactions with immune cells in the tumor microenvironment (TME). They can cause DNA damage *via* metabolites, participate in signaling pathways, and modulate immune responses [1]. Some microbes promote Treg and myeloid-derived suppressor cell proliferation, thereby supporting tumor growth [2]. Receptor-interacting serine/threonine-protein kinase 2 (Ripk2) plays a crucial role in mediating inflammatory and innate immune signals [3]. Nod1/2 is capable of recognizing muramyl dipeptide fragments in bacterial cell walls, which recruit Ripk2 through the CARD domain to activate inflammatory and immune responses in the organism [4]. Although Ripk2 is linked to tumor proliferation, metastasis, and drug resistance, and is known to regulate the TME, its specific role in the HCC immune microenvironment remains unclear.

In the tumor immune microenvironment, Macrophages and CD8^+^ T cells engage in complex cross-talk crucial for tumor progression and immune responses. Macrophages can activate CD8^+^ T cells by presenting antigens and secreting chemokines. However, they can also inhibit CD8^+^ T cell function by polarizing to the M2 phenotype, secreting immunosuppressive cytokines, or expressing PD-L1 [5, 6]. In HCC, M1 macrophages promote diethylnitrosamine (DEN)-induced precancerous lesions [7]. Ripk2 is highly expressed in monocytes/macrophages and correlates with immunotherapy response [8]. While macrophage-derived Ripk2 promotes osteosarcoma invasion [9], its role in HCC progression is unknown.

This study aimed to elucidate the interactions and mechanisms among intratumoral microbiota, Ripk2, and macrophages in HCC. Using animal models, scRNA-seq, RNA-seq, co-culture assays, and molecular techniques, we provide a theoretical foundation for novel therapeutic strategies.

## Materials and methods

### Compounds, reagents and kits

Diethylnitrosamine (DEN) (CAS: 55-18-5) was obtained from Shanghai Acmec Biochemical Co., Ltd. GSK-583 (CAS: HY-100339), Cxcl9 (74-103) (CAS: HY-P10301), BMS202 (CAS: HY-19745) and AMG 487 (CAS: HY-15319) were obtained from MedChemExpress LLC. Clodronate liposomes (CAS: 40337ES08) were obtained from Yeasen Biotechnology (Shanghai) Co., Ltd. The sources of other compounds and reagents are as follows: Collagenase from Clostridium histolyticum, type IV (Collagenase IV) (Sigma-Aldrich, CAS: C5138), Deoxyribonuclease I from bovine pancreas (DNase I) (Sigma-Aldrich, CAS: DN25), Tumor Dissociation Kit (Miltenyi, CAS: 130-096-730), CD45 (TIL) MicroBeads (Miltenyi, CAS: 130-110-618), Cell Staining Buffer (Biolegend, CAS: 420201), Phosphate-buffered saline (PBS) (Solarbio, CAS: P1020), Hematoxylin-Eosin (HE) Stain Kit (Solarbio, CAS: G1120), Percoll (Sigma-Aldrich, CAS: GE17-0891), Dead Cell Removal Kit (Miltenyi, CAS: 30-090-101), TruStain FcX™ (anti-mouse CD16/32) Antibody (Biolegend, CAS: 101319), anti-Ripk2 (Affinity, CAS: DF6967), anti-p-Ripk2 (Affinity, CAS: AF0049), anti-Adpgk (Affinity, CAS: DF3414), anti-Cxcl9 (Proteintech, CAS: 22355-1-AP), anti-Sox6 (Santa Cruz Biotechnology, CAS: sc-393314), anti-Myb (Santa Cruz Biotechnology, CAS: sc-74512), anti-GAPDH (Proteintech, CAS: 60004-1-Ig), anti-Tubulin (Cell Signaling Technology, CAS: 2146).

### Clinical specimens

The Fujian Cancer Hospital generously supplied HCC tissue samples for this study, following approval granted by the Institutional Ethics Committee of Fujian Cancer Hospital. The staging and grading of the tumors were conducted in accordance with the eighth edition of the American Joint Committee on Cancer criteria, and histopathology was used to confirm the diagnosis of HCC.

### Animal

Male C57BL/6 J mice, aged six to eight weeks and weighing approximately 17-21 grams, were procured from Shanghai SLAC Laboratory Animal Co., Ltd. To construct Ripk2 knockout mice (Ripk2^*CKO*^), Ripk2^*flox/flox*^ mice (T051882, GemPharmatech) were crossed with Lyz2-iCre transgenic mice (T003822, GemPharmatech) according to the experimental protocol. All animals were maintained in a specific pathogen-free (SPF) environment, with the temperature and humidity controlled at 21–23 °C and 40–55%, respectively. The animal experiments were conducted in accordance with the guidelines of the Institutional Animal Care and Use Committee and were approved by the Experimental Animal Ethics Committee of Fujian Medical University (IACUC FJMU 2025-0112).

### Establishment and treatment of subcutaneous HCC model

To establish a subcutaneous tumor model, six-week-old male C57BL/6 J mice were randomly distributed into distinct experimental groups. The Hep53.4 cells (5×10^6^/mL) were then injected subcutaneously into the right flank of each mouse. On the 10th day, mice were intraperitoneally injected with *Sc* (purchased from Mingzhoubio, Ningbo, China). On the fifth day of *Sc* treatment, BMS202 (10 mg/kg, once every 2 days, a total of 6 injections) was administered alone or in combination with GSK583 (5 mg/kg, once every 2 days, a total of 4 injections). On the 15th day of treatment, 0.3% pentobarbital sodium solution was injected intraperitoneally to induce anesthesia in mice. Then, the mice were euthanized humanely by cervical dislocation.

### Construction and treatment of orthotopic HCC model

The DEN-induced orthotopic HCC model was based on our previous research [10]. 4-week-old male C57BL/6 J and Ripk2^*CKO*^ mice were intraperitoneally injected with DEN (50 mg/kg) once a week for 32 weeks. Subsequently, AMG 487 (5 mg/kg) or GSK583 (5 mg/kg) was administered intraperitoneally once every 3 days, for a total of 4 injections.

### Expression and prognostic analysis of Ripk2

We obtained data from the TCGA database (https://portal.gdc.cancer.gov) by downloading STAR counts data and accompanying clinical information pertaining to liver cancer. Subsequently, we extracted the data in TPM format and normalized it using the formula log2 (TPM + 1). Following filtering, we selected samples that had both RNA-seq data and clinical information.To construct the Kaplan-Meier curve, we conducted a log-rank test to determine the *P*-value. We utilized univariate Cox regression to calculate the hazard ratio (HR) and its corresponding 95% confidence interval. For statistical analysis, we employed R software, version 4.0.3. We considered results to be statistically significant when the HR value exceeded 1 and the *P*-value was less than 0.05, suggesting a robust correlation between the studied factors and patient prognosis.

### Cell lines

The murine HCC cell line Hep53.4 (BioVector NTCC Inc., Cat no: CVCL_5765) was cultured in Dulbecco’s modified eagle medium (DMEM) supplemented with 10% fetal bovine serum (FBS) and 100 U/ml penicillin/streptomycin. The cells were maintained at 37 °C in a 5% CO₂ incubator to ensure standardized growth conditions. Routine mycoplasma contamination tests were conducted on the cell lines, confirming their freedom from mycoplasma contamination.

### Plasmids

Plasmids HA-Sox6, HA-Myb and GFP-Myb were obtained from Tsingke Biotechnology Co. Ltd.

### Establishment of cells

The human or mouse Ripk2 overexpression (OE) plasmid was obtained from Tsingke Biotechnology Co., Ltd. Subsequently, BMDMs or THP-1 cells were transfected with plasmids. After a 48-h incubation, the cells were further selected using a two-day culture containing 3 μg/ml puromycin.

### siRNA and shRNA transfection

siRNA targeting mouse Myb, Sox6 and Pax5, and shRNA targeting human Ripk2 were obtained from Tsingke Biotechnology Co., Ltd. shRNA was transfected into cells using Lipofectamine 3000. After 48 h, we used Western blotting to detect the protein expression levels of Myb, Sox6 and Pax5.

### Extraction and cultivation of bone marrow-derived macrophages (BMDMs)

In brief, mice were anesthetized with sodium pentobarbital and subsequently dissected under sterile conditions using 75% ethanol for disinfection. Bone marrow was extracted by flushing the bones with RPMI 1640 through a syringe. The harvested cells were then filtered and cultured. On the subsequent day, the mononuclear cell layer was isolated and cultured in RPMI 1640 enriched with 10% fetal bovine serum. Differentiation of these cells into macrophages was induced by supplementing the culture medium with 50 ng/mL of macrophage colony-stimulating factor (GM-CSF; CAS: 91108ES08, Yeasen Biotechnology Co., Ltd.). The culture media were replaced on the third and fifth days, and by the seventh day, the bone marrow-derived macrophages (BMDMs) had matured.

### Co-cultivation system

We co-cultured BMDMs with mouse spleen-derived T cells. In short, IL-2 was used to maintain the activity of mouse spleen-derived T cells. T cells (1×10^6^ cells/mL) were seeded in the lower layer of the chamber, while BMDMs (1×10^6^ cells/mL) (with or without Ripk2 knockout) were seeded in the upper layer. After co-culturing for 24 h, AMG 487 (5 μM) was used to stimulate T cells. After 24 h, T cells were collected from the lower layer for subsequent analysis.

Establishment of a co-culture system of T cells and Hep53.4 cells. In brief, the Hep53.4 cells (5×10^5^ cells/mL) were resuspended in the culture medium and inoculated into 24-well culture plates with a total volume of 1 mL. In the upper layer of the chamber, activated T cells were added at a 1:1 ratio, with a total volume of 500 μL. After incubation for 4 h, Cxcl9-derived peptide (2 μM) was added to the upper chamber. After 18 h of continuous culture, Hep53.4 cells in the lower chamber were collected for apoptosis analysis.

Gene-edited THP-1 cells were co-cultured with human T cells in vitro. In brief, peripheral blood mononuclear cells from healthy individuals were cultured in T cell culture medium supplemented with IL-2 (10 ng/mL) and human CD3/CD28/CD2 T cell activator (25 μg/mL) for 5 days. Inoculate gene-edited THP-1 cells (5×10^5^ cells/mL) into the upper layer of the chamber. Add activated T cells to the lower layer of the chamber in a 1:1 ratio between THP-1 cells and activated T cells, with a total volume of 500 μL, after 36 h of incubation. Collect T cells in the lower chamber. FCM was used to detect the expression of IFN-γ (Cat 383303; Biolegend).

### CFSE cell tracer assay

We labeled the T cells using CFSE (C0051, Beyotime, Shanghai, China) and inoculated them into a six-well plate for an overnight period. Subsequently, the cells were washed with PBS, and resuspended. Thereafter, the fluorescence intensity of the cells was measured meticulously using a FACS Celesta™ flow cytometer (BD Biosciences, NJ, USA).

### Peritumoral injection of gene-transfected BMDMs

C57BL/6 J mice were subcutaneously injected with Hep53.4 cells (1×10^6^/mL). After 10 days, the mice were randomly divided into 2 groups, with 6 mice in each group. BMDM/Ripk2-OE cells (1×10^6^/mL) and BMDM/OE-NC cells (1×10^6^/mL) were injected around the tumor, respectively. After 15 days, we isolated the tumor tissue from the mice and calculated the tumor size.

### Chromatin Immunoprecipitation (ChIP)

For chromatin immunoprecipitation (ChIP), cells were first cross-linked with formaldehyde to fix protein–DNA interactions. Chromatin was then sheared into fragments by sonication. Target proteins (Myb or Sox6) were immunoprecipitated using specific antibodies coupled to magnetic beads. After extensive washing to remove nonspecific binding, cross-links were reversed and enriched DNA was purified for downstream analysis. These purified fragments were then subjected to PCR analysis to elucidate the protein-DNA interactions. The primers utilized in the PCR were sourced from Tsingke Biotechnology Co., Ltd., with the following sequences: F_Myb: GATAGGAAACTGTAGCCACGG; R_Myb: GAAGGTTGGAGATGTACTTCCCAC; F_Sox6 (+): CTAGTCGTGACATATCAAAGGAC; R_Sox6 (+): GATCCACGTACACAAAACAACAG; F_Sox6_2 (-): GCCGTCTCTAGAGGAGATAAG; R_Sox6_2 (-): CATTGAGTGCACTCATTAATAGAAG. The PCR amplification protocol consisted of an initial pre-denaturation step at 95 °C for 5 min, followed by 40 cycles of denaturation at 95 °C for 30 s, annealing at 50 °C for 30 s, and extension at 72 °C for 10 s.

### Dual luciferase reporter (DLR)

The pGL3-Cxcl9-luc, pGL3-Mut-Cxcl9-luc and pGL3-Adpgk-luc were acquired from Tsingke Biotechnology Co. Ltd. The cells were subsequently co-transfected with this reporter plasmid and the pGL3-basic vector, utilizing Lipofectamine 3000 as the transfection reagent. Furthermore, pRL-TK, sourced from Promega, was included in the transfection mixture to serve as an internal control. After transfection, the cells were incubated for 36 h. Upon completion of this incubation period, cell lysates were meticulously prepared and subjected to a dual-luciferase assay, adhering strictly to the manufacturer’s guidelines provided by Promega.

### Seahorse assay

Cellular glycolysis was assessed with an XF96 extracellular flux analyzer (Seahorse Bioscience). Cells were seeded in XF96 microplates at 12,000 cells per well (~90% confluence). Prior to analysis, the medium was replaced with unbuffered assay medium, and cells were equilibrated for 1 h at 37 °C in a non‑CO₂ incubator. The extracellular acidification rate (ECAR) was measured at baseline and after sequential injection of metabolic modulators. ECAR values, expressed in mpH/min, were normalized to total protein content.

### Immunohistochemistry (IHC)

For IHC assays, all sections underwent a boiling process in sodium citrate buffer for 10 min, followed by a 20 min incubation period in 3% hydrogen peroxide. Subsequently, the sections were blocked with 1% goat non-immune serum for 60 min. Following this, they were incubated with primary antibodies specifically targeting Ripk2 (Affinity, CAS: DF6967, diluted at 1:100) at a temperature of 37 °C for an additional 60 min. After incubation with horseradish peroxidase (HRP)-conjugated secondary antibodies supplied by Affinity Biosciences, the sections were developed using 3,3′-diaminobenzidine.

### Immunoblotting

In brief, 30 μg of total protein samples were resolved by SDS-PAGE and then transferred onto PVDF membranes (Biosharp, Hefei, China). Following this, the membranes were blocked with 5% bovine serum albumin (BSA) for 1 h at room temperature. The membranes were then incubated with primary antibodies overnight at 4 °C. Subsequently, secondary antibodies were introduced, and the membranes were incubated for another hour at room temperature. The protein bands were detected and visualized utilizing a Hypersensitive ECL Chemiluminescence Kit (NcmECL Ultra, ABP Biosciences, Beltsville, MD, USA). The primary antibodies employed in this study included anti-Ripk2 (dilution 1:1000), anti-p-Ripk2 (dilution 1:1000), anti-Tubulin (dilution 1:1000), anti-Cxcl9 (dilution 1:1000), anti-Myb (dilution 1:1000) and anti-Adpgk (dilution 1:1000).

### Mouse serum analyses

Serum was separated by centrifugation at 4000 rpm for 20 min. The serum levels of alanine aminotransferase (ALT) and aspartate aminotransferase (AST) were determined using an assay kit (Jiancheng Bioengineering Institute, Nanjing, China) according to the manufacturer’s instructions. Serum Cxcl9 levels were measured using commercially available enzyme-linked immunosorbent assay (ELISA) kits (Elabscience Bio) following the manufacturer’s instructions.

### Fluorescence in situ hybridization (FISH) assay

The tissue sections underwent a washing process in 0.1 M Tris-HCl buffer, adjusted to a pH of 7.4, for a period of 15 min. The *Sc*-specific FISH probe (G16F67-taxID42684) was obtained from GeWei Bio-Tech (Shanghai) Co. Ltd. and fluorescently labeled with the texasred fluorophore, which has an excitation wavelength of 590 nm and an emission wavelength of 617 nm. Subsequently, the presence of *Sc* colonization within the tissues was visualized and analyzed using a Leica TCS SP8 microscope.

### RNA-seq

The RNA libraries were sequenced by OE Biotech, Inc., Shanghai, China. Total RNA was extracted from each sample employing an RNA mini kit sourced from Qiagen, Germany. The quality of the RNA was assessed through gel electrophoresis and a Qubit fluorometer. Differential expression analysis between the two groups was conducted using the DESeq2 R package. Subsequently, differentially expressed transcripts (DETs) were selected for in-depth functional and signaling pathway enrichment analysis, leveraging the Gene Ontology (GO) and Kyoto Encyclopedia of Genes and Genomes (KEGG) databases.

### Flow Cytometry (FCM)

After meticulously digesting tumor tissues with the tumor dissociation kit, lymphocytes were isolated for subsequent analysis. To deplete erythrocytes, 2 mL of red blood cell lysis buffer was added into the cell suspension, which was then incubated for 15 min at room temperature. Subsequently, 30 mL of ficoll plus (#P4350, Solarbio, Beijing, China) was added, and the cell suspension was carefully layered onto the surface of the separation medium. The mixture was centrifuged at 900×g for 30 min at room temperature to segregate the lymphocyte layers. For immunostaining preparation, the cell concentration was adjusted to 2×10^5^/mL using cell staining buffer. Subsequently, fluorescently labeled primary antibodies were added into the cell suspension and incubated for 30 min at 4 °C in a dark environment to ensure specific antibody binding. A FACS Celesta™ Flow Cytometer (BD Biosciences, NJ, USA) was employed to detect the expression of target proteins on the lymphocyte surface. The antibodies used for characterizing CD8^+^T and macrophages cells comprised BV605-Fixable Viability Stain (BD Pharmingen), BV650-F480 (BM8, BioLegend, San Diego, CA), APC-CD206 (MR6F3, eBioscience, San Diego, CA), APC/Cy7-CD3 (17A2, BioLegend), FITC-CD45 (QA17A26, BioLegend), PerCP/Cyanine5.5-CD8 (53-6.7, BioLegend), PE-GZMB (QA16A02, BioLegend), BV421-CD4 (GK1.5, BioLegend), and PE/Cy5-CD80 (16-10A1, BioLegend).

### scRNA-seq

scRNA-seq was performed by 10 K Genomics (Shanghai, China). Briefly, immune cells were isolated from tumors of mice in three groups (WT, GSK583-treated, and Ripk2^*CKO*^; *n* = 3 per group). Single-cell suspensions were used to prepare libraries with the 10× Genomics Chromium™ Single Cell 3’ Reagent Kit. Within the system, cells were encapsulated in droplets with gel beads carrying unique primers containing a cell barcode, UMI, and poly(dT) sequence. Following reverse transcription to produce barcoded cDNA, the emulsion was broken and cDNA was amplified. The sequencing library was constructed through cDNA fragmentation, end repair, adapter ligation, and size selection. Final libraries were sequenced on an Illumina NovaSeq 6000 platform (paired-end, 150 bp).

### Statistical analysis

All experiments have at least three independent replicates. The comparison between the two groups was conducted using a t-test. Multiple groups were analyzed using ANOVA. Statistical analyses were performed using SPSS version 25.0 software. All data were presented in the form of mean ± SD.

## Results

### Ripk2 is highly expressed in HCC and correlates with poor prognosis

**Fig. 1:**
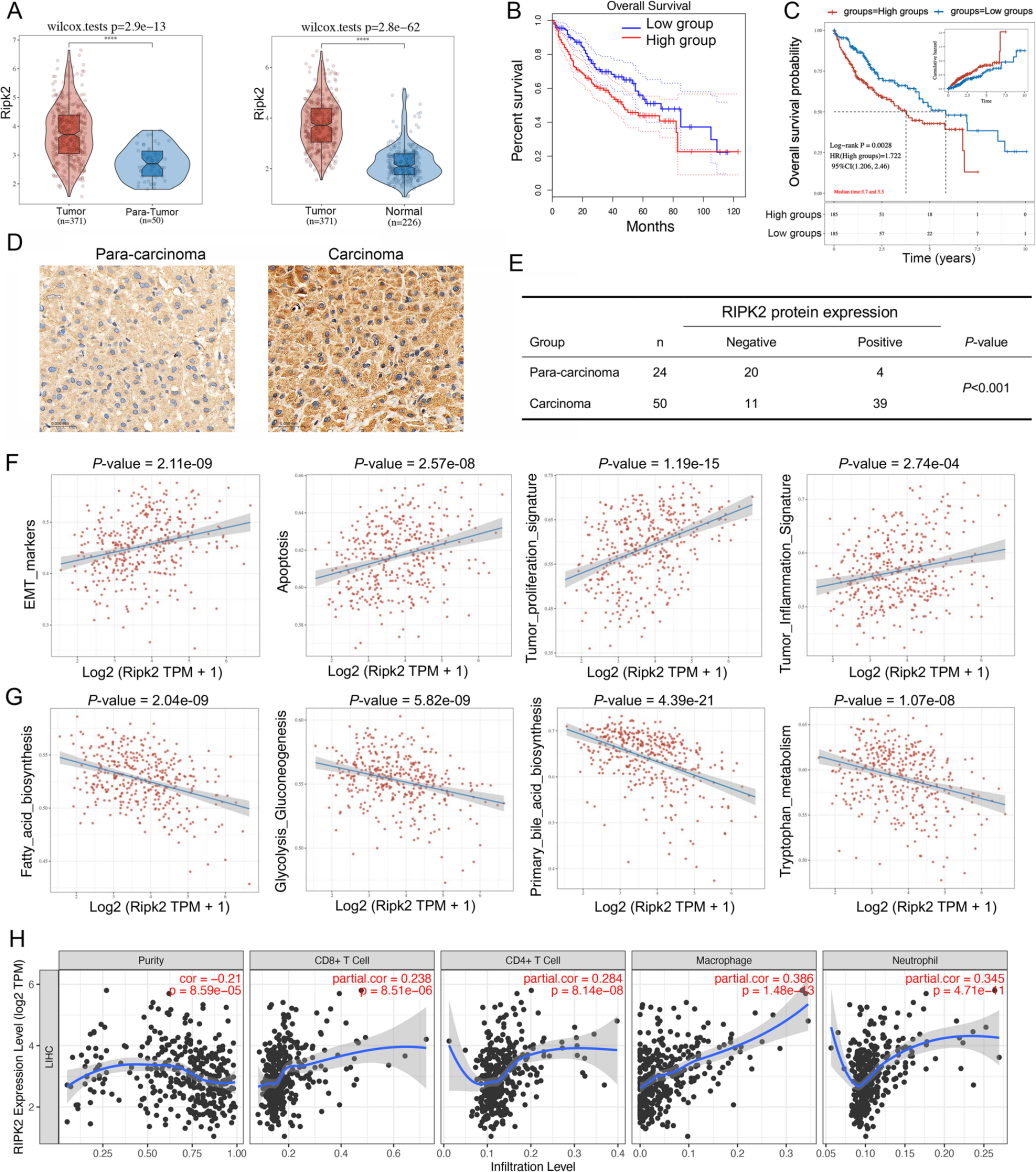
Ripk2 is overexpressed in human HCC and correlates with low patient survival rates. **A** We compared Ripk2 expression levels in LIHC cancer tissues (*n* = 371) with adjacent non-cancerous samples (*n* = 50) and normal liver tissue samples (*n* = 226) from the TCGA database. **B** The relationship between Ripk2 and prognostic survival in liver cancer patients was analyzed using the GEPIA2.0 online website. **C** Ripk2 was divided into high and low expression groups, and the relationship between its expression and prognostic survival in liver cancer patients was analyzed. **D** Immunohistochemical detection of Ripk2 expression in carcinoma and para-carcinoma. **E** Chi-square test analysis of Ripk2 expression differences. Adjacent tissues (para-carcinoma, *n* = 24), cancer tissues (carcinoma, *n* = 50). **F** Correlation between Ripk2 gene expression and EMT, apoptosis, tumor proliferation and inflammation. **G** Correlation between Ripk2 gene expression and fatty acid synthesis, glucose metabolism, bile acid synthesis and tryptophan metabolism. **H** We used the TIMER online database to analyze the relationship between Ripk2 gene expression and the infiltration of immune cells (CD8^+^ T, CD4^+^ T, macrophages and neutrophils) in HCC.

We found that Ripk2 expression was significantly elevated in cancer tissues compared to both adjacent non-cancerous samples and normal liver tissues by analyzing TCGA-LIHC data (Fig. 1A). High Ripk2 expression was associated with worse patient survival (Fig. 1B, C). Immunohistochemistry confirmed higher Ripk2 levels in tumor tissues (Fig. 1D, E). To clarify the relationship between Ripk2 gene expression and biological functions, we conducted correlation analysis and found that Ripk2 gene expression positively correlated with epithelial-mesenchymal transition (EMT), apoptosis, tumor proliferation and inflammation (Fig. 1F), while negatively correlating with cellular metabolic signals such as fatty acid biosynthesis, glycolysis, bile acid synthesis, and tryptophan metabolism (Fig. 1G). These findings suggest that Ripk2 may negatively impact metabolism and promote HCC. Additionally, we used the TIMER online database to analyze the relationship between Ripk2 gene expression and immune cell infiltration in HCC. Ripk2 gene expression was positively correlated with the infiltration of CD8^+^ T, CD4^+^ T, macrophages, and neutrophils, with the strongest correlation observed with macrophages (Fig. 1H). In addition, the expression level of Ripk2 was positively correlated with the infiltration level of F4/80^+^ macrophages in tumor tissues by analyzing HCC clinical samples (Fig. S1), suggesting a significant interaction between Ripk2 and macrophages in HCC.

### Ripk2 inhibition increases Streptomyces collinus abundance in macrophages

**Fig. 2:**
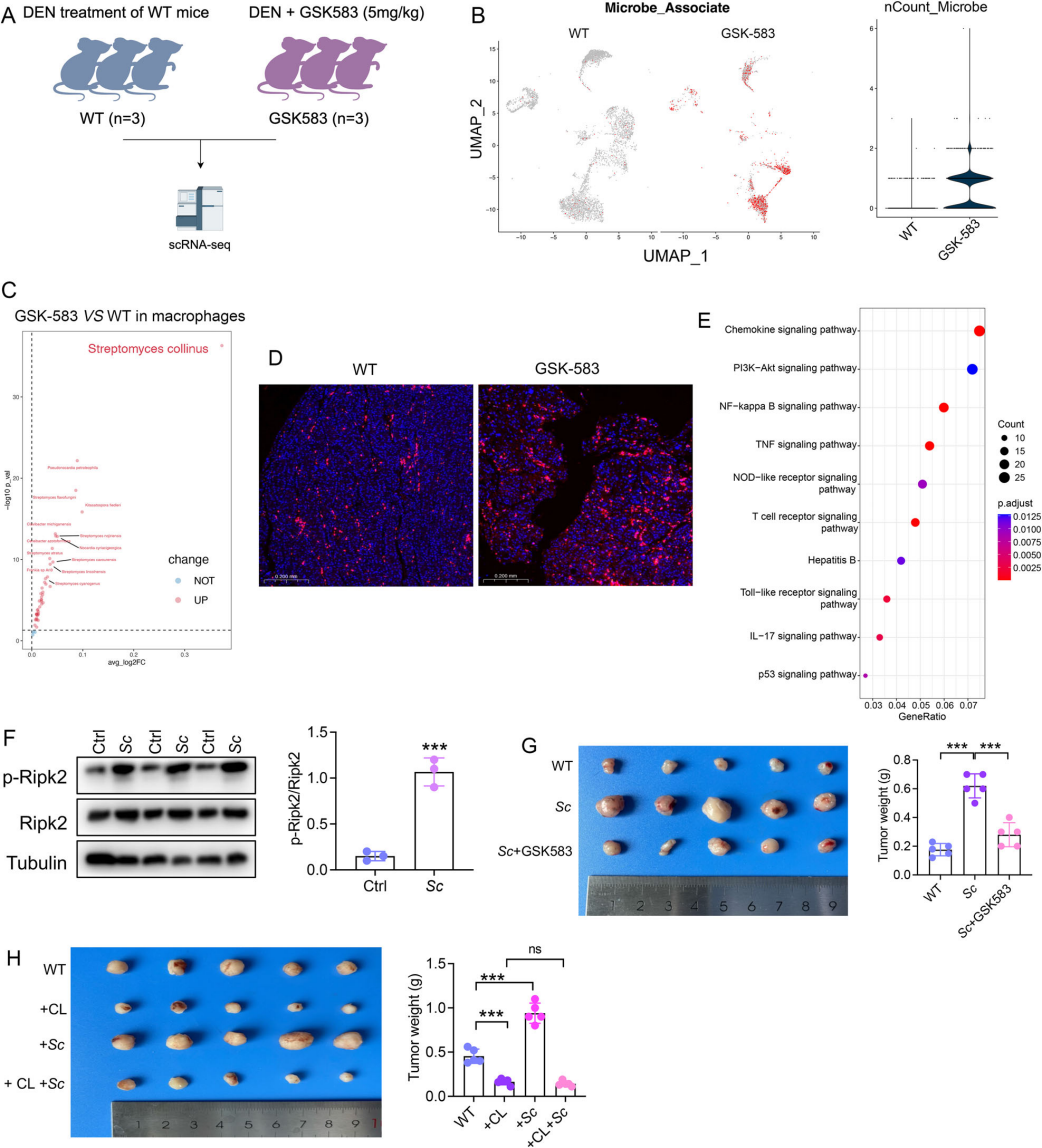
Ripk2 inhibitor enhances the proportion of microbe in macrophages. **A** The scRNA-seq analysis of DEN induced primary HCC with and without GSK583 treatment. *n* = 3. **B** The scRNA-seq analysis of the proportion of microbe within macrophages. Comparison of microbial quantities between the wild-type (WT) and Ripk2 inhibitor group (GSK583). **C** Top 10 microbe upregulated in the GSK583 group compared to the WT group. **D** FISH experiments. *n* = 3. **E** KEGG enrichment analysis of signaling pathways associated with the upregulated microbe. **F** Immunoblotting was used to analyze the levels of Ripk2 and p-Ripk2 in macrophages treated with *Sc*. *n* = 3. Data were mean ± SD and analyzed by t test. **G** The effect of peritumoral injection of *Sc* on the growth of HCC with or without GSK583 treatment. *n* = 5. Data were mean ± SD and analyzed by ANOVA. **H** We used clodronate liposomes (CL) to clear macrophages in mice. The effect of peritumoral injection of *Sc* on the growth of HCC with or without elimination of macrophages. *n* = 5. Data were mean ± SD and analyzed by ANOVA. “ns” means no significant difference. ***P < 0.001.

Macrophages eliminate infections by specifically recognizing and phagocytosing pathogens while also mediating inflammatory responses. To explore the relationship between Ripk2 and macrophages within the immune microenvironment of HCC, we treated DEN-induced primary HCC with the Ripk2 inhibitor (GSK583) (Fig. 2A). scRNA-seq analysis revealed that GSK583 increased the proportion of microbiota within macrophages (Fig. 2B), with *Streptomyces collinus* (*Sc*) showing the most significant increase (Fig. 2C). FISH experiments confirmed elevated *Sc* abundance in GSK583-treated tumors (Fig. 2D). By isolating macrophages from tumor tissues, it was found that macrophages in the GSK583 treatment group phagocytized more *Sc* (Fig. S2). Pathway enrichment analysis of Sc-associated genes highlighted Nod-like and Toll-like receptor signaling (Fig. 2E, Table S1). In vitro experiments confirmed that *Sc* can activate the Ripk2 signaling in macrophages (Fig. 2F). Additionally, Peritumoral injection of *Sc* promoted HCC growth, an effect blocked by GSK583 (Fig. 2G), suggesting that *Sc* promotes HCC growth through Ripk2 signaling. Depleting macrophages also abolished the tumor-promoting effect of *Sc* (Fig. 2H). These findings establish a close relationship between *Sc*, Ripk2, and macrophages in HCC.

### Macrophage-specific Ripk2 knockout inhibits HCC growth

**Fig. 3:**
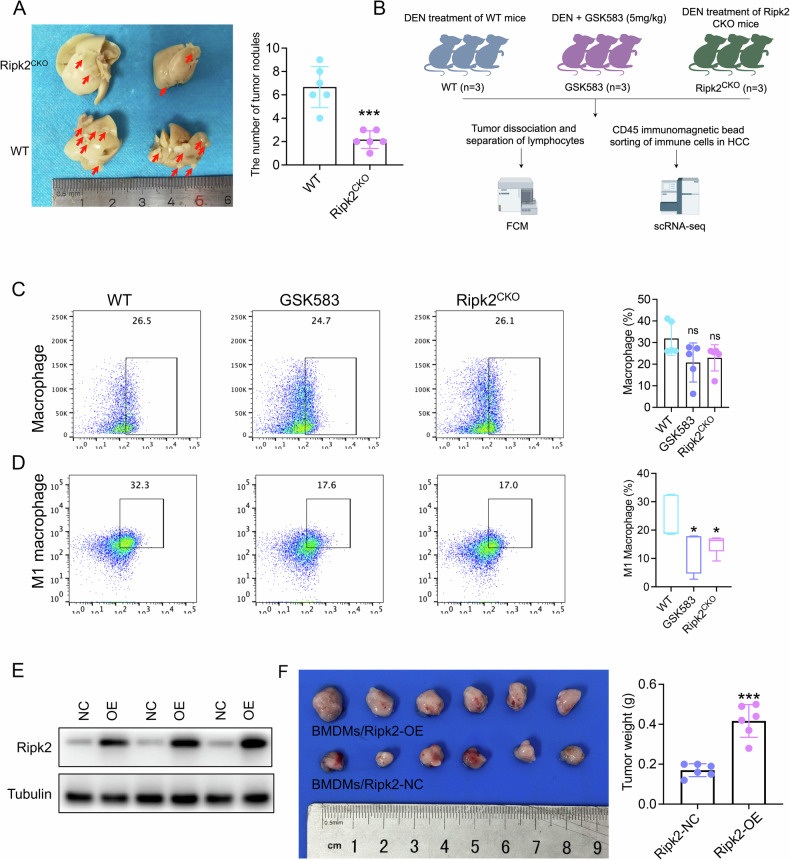
Knockout of Ripk2 in macrophages inhibits HCC growth. **A** The effect of Ripk2 knockout of macrophages on HCC growth. *n* = 6. Data were mean ± SD and analyzed by t test. **B** Schematic diagram illustrating the in-depth analysis using FCM and scRNA-seq in the context of established primary HCC. *n* = 3. **C,** **D** FCM analysis of the proportion of macrophages and M1 macrophages in HCC tissues from WT, GSK583-treated, and Ripk2^*CKO*^ groups. *n* = 5. Data analysis was performed using one-way ANOVA. **E** Immunoblotting was used to detect the expression level of Ripk2. *n* = 3. “NC” represents the control group, and “OE” represents overexpression of Ripk2. Data were mean ± SD and analyzed by t test. **F** Injection of established BMDM/Ripk2-OE and BMDM/Ripk2-NC cells into the peritumoral subcutaneous tissue to analyze the impact of macrophage Ripk2 on HCC growth. *n* = 6. Data were mean ± SD and analyzed by *t* test. “ns” means no significant difference. *P < 0.05, ***P < 0.001.

To elucidate the role of macrophage-derived Ripk2 in the progression of HCC, we employed mice with Ripk2 knockout in macrophages (Ripk2^*CKO*^). The number of tumor nodules in Ripk2^*CKO*^ mice was significantly smaller compared to wild-type (WT) mice (Fig. 3A). Treatment of DEN-induced HCC with GSK583 in Ripk2^*CKO*^ and WT mice showed that Ripk2 deletion or inhibition did not alter total macrophage proportions but reduced M1 macrophage numbers (Fig. 3B–D). Furthermore, we isolated bone marrow-derived macrophages (BMDMs) from mice and established a BMDM/Ripk2-OE cell line. We also detected the expression level of Ripk2 through immunoblotting (Fig. 3E). Intratumoral injection of BMDM/Ripk2-OE cells significantly promoted HCC growth (Fig. 3F). These results suggest that macrophage-derived Ripk2 promotes HCC progression.

### Ripk2 shapes the immune microenvironment in HCC

**Fig. 4:**
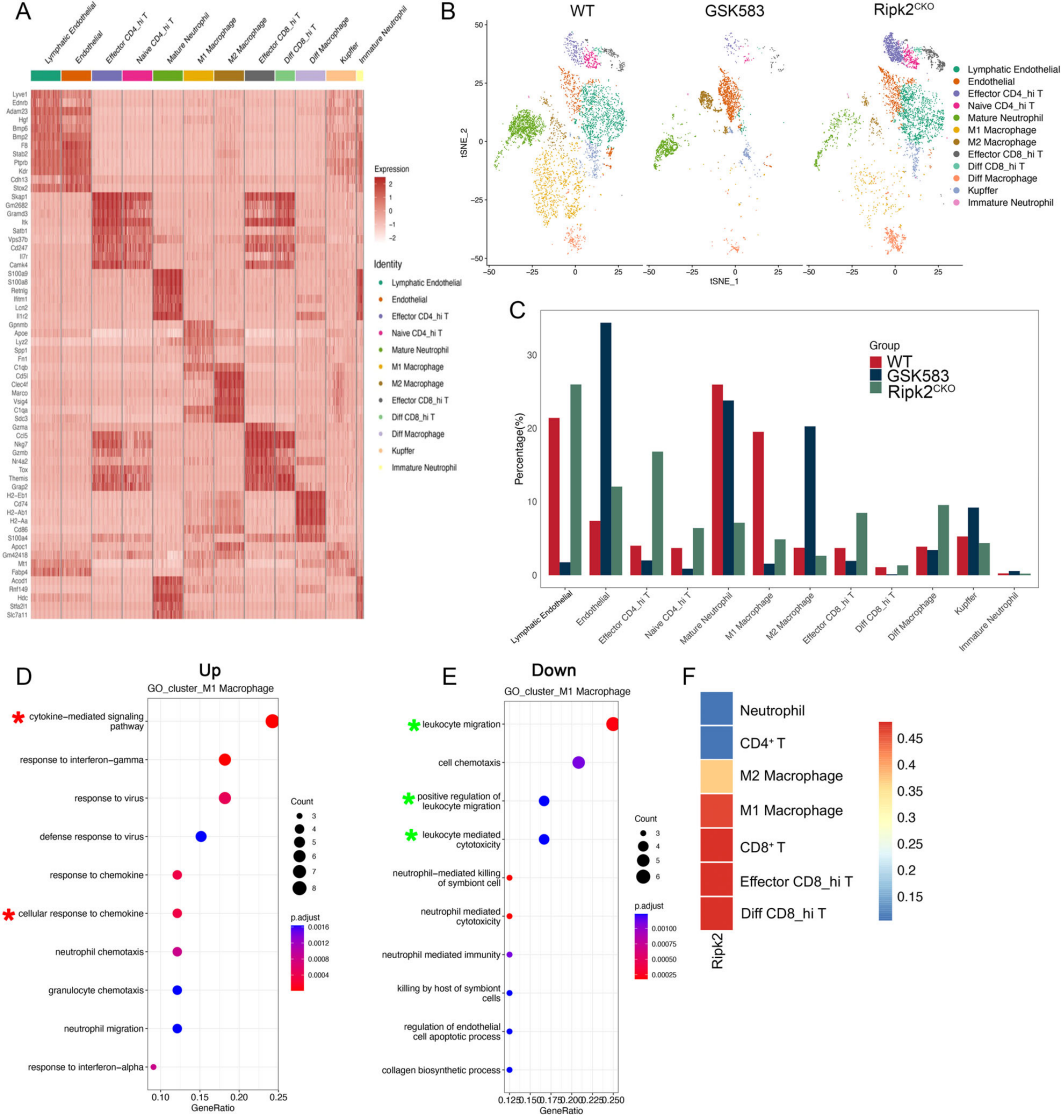
Ripk2 influences the cellular composition of the immune microenvironment in HCC. **A** Heatmap of gene clustering. **B** Cell island plot obtained from dimensionality reduction analysis using tSNE for the WT, GSK583, and Ripk2^*CKO*^ groups. *n* = 3. **C** Proportion plot of immune cells. **D,** **E** GO enrichment analysis of commonly upregulated or downregulated genes in the GSK583 and Ripk2^*CKO*^ groups. **F** Correlation between Ripk2 expression and immune cell infiltration.

To analyze the impact of macrophage-derived Ripk2 on the immune microenvironment of HCC, we categorized cells identified by scRNA-seq into lymphatic endothelial, endothelial, effector CD4_hi T, naive CD4_hi T, mature neutrophil, M1 macrophage, M2 macrophage, effector CD8_hi T, diff CD8_hi T, diff macrophage, kupffer, and immature neutrophil (Fig. 4A). Compared to the WT group, both the GSK583 and Ripk2^*CKO*^ groups showed a significant increase in the number of endothelial cells, while also markedly decreasing the number of mature neutrophils and M1 macrophages (Fig. 4B, C). To elucidate the effect of Ripk2 on M1 macrophages, we conducted GO enrichment analysis on the commonly upregulated and downregulated genes. The upregulated genes were primarily enriched in cytokine-mediated signaling pathways and cellular responses to chemokines (Fig. 4D). In contrast, the downregulated genes were mainly concentrated in leukocyte migration and leukocyte-mediated cytotoxicity (Fig. 4E), suggesting that Ripk2 influences M1 macrophages by modulating cytokine/chemokine secretion. Correlation analysis confirmed Ripk2 expression was most closely linked to M1 macrophages and CD8^+^ T cell infiltration (Fig. 4F). These data emphasize that macrophage-derived Ripk2 affects the cellular composition of the immune microenvironment in HCC.

### Ripk2 knockout induces CD8^+^T cell infiltration via Cxcl9 activation

**Fig. 5:**
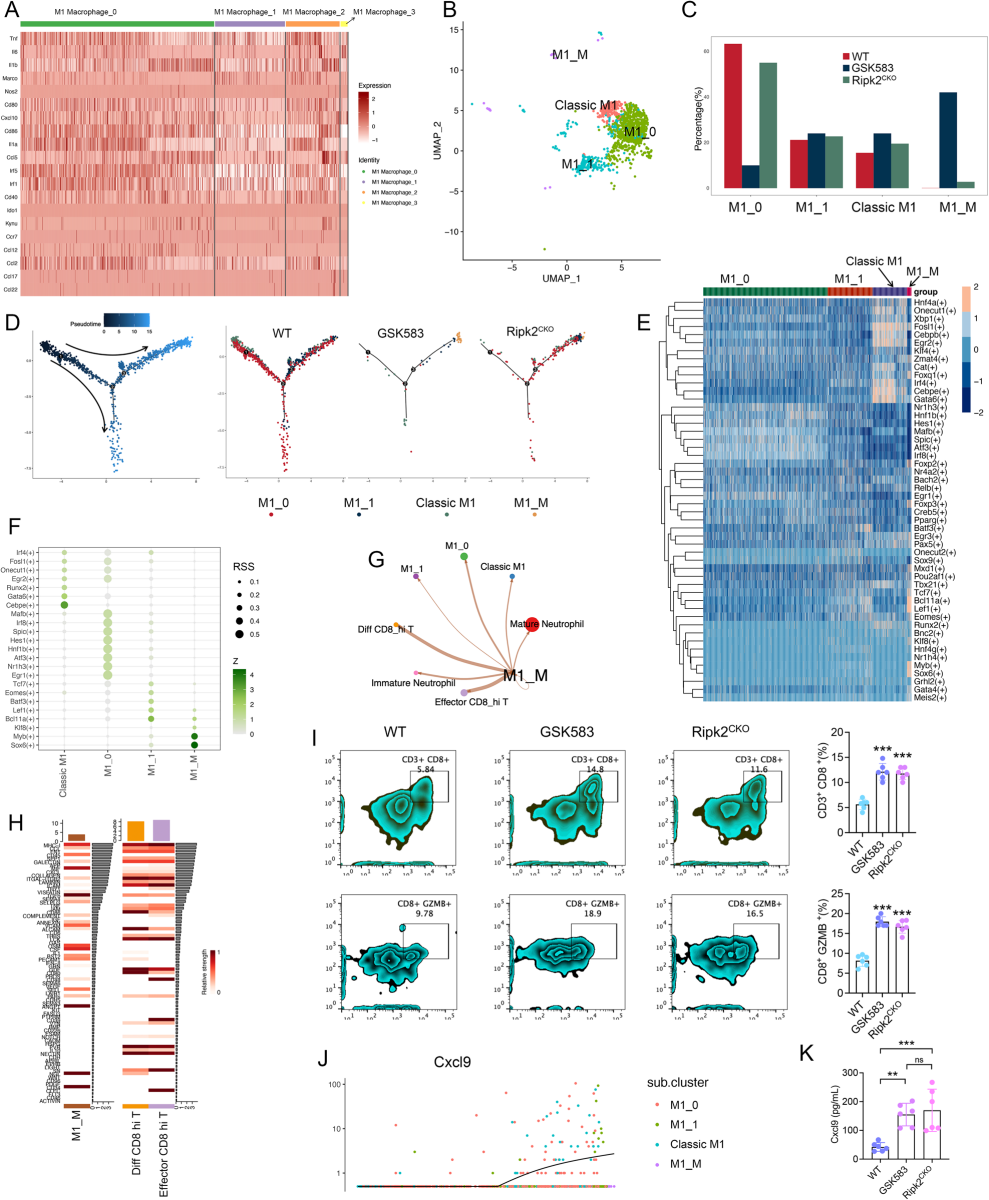
Inhibition of macrophage Ripk2 induces CD8^+^T cell recruitment in HCC by activating Cxcl9. **A** Heatmap of gene clustering. **B** Cell island plot obtained from dimensionality reduction analysis using UMAP for M1 macrophages. *n* = 3. **C** Proportions of M1 macrophage subpopulations. **D** Pseudotime trajectory analysis. **E,** **F** Heatmap of regulon analysis and specific scores. **G** Cell communication analysis. **H** Heatmap of cell receptor-ligand interactions. **I** FCM analysis of the impact of Ripk2 on the proportions of CD3^+^CD8^+^ cells and CD8^+^GZMB^+^ cells. *n* = 6. Data were mean ± SD and analyzed by one-way ANOVA. **J** Cxcl9 gene expression analysis along the pseudotime trajectory. **K** ELISA detection of serum Cxcl9 secretion levels. *n* = 6. Data were mean ± SD and analyzed by ANOVA. “ns” means no significant difference. **P < 0.01, ***P < 0.001.

We identified four distinct subclusters within the M1 macrophage population, designated as M1 macrophage_0, M1 macrophage_1, M1 macrophage_2, and M1 macrophage_3 (Fig. 5A). Based on the highly variable genes of each subpopulation, we defined M1 macrophage_2 as the classic M1 macrophage (Classic M1) and M1 macrophage_3 as the metabolic M1 macrophage (M1 metabolism, M1_M) (Fig. 5B). Notably, the proportions of M1_M in both the Ripk2^*CKO*^ and GSK583 groups were significantly higher than those in the WT group (Fig. 5C). Additionally, we employed pseudotime trajectory analysis and discovered that M1 macrophages in both the Ripk2^*CKO*^ and GSK583 groups predominantly evolved towards M1_M (Fig. 5D). We next conducted an in-depth analysis of the functions and characteristics of M1_M, as well as its interactions with other immune cells. Regulon scores indicated that Myb and Sox6 were specific regulons for M1_M (Fig. 5E, F). Cell communication analysis suggested that M1_M was most closely related to diff CD8_hi T and effector CD8_hi T cells (Fig. 5G). M1_M interacted with diff CD8_hi T and effector CD8_hi T cells mainly through cytokines, chemokines, and adhesion molecules such as Ccl, Mif, Itgal, Icam, Tgfb and Alcam (Fig. 5H). FCM detection revealed that the proportions of CD3^+^ CD8^+^ cells and CD8^+^ GZMB^+^ cells were significantly higher in the Ripk2^*CKO*^ and GSK583 groups compared to the WT group (Fig. 5I), indicating that targeting Ripk2 in macrophages could activate the killing activity of CD8^+^ T cells. To further validate our hypothesis, we constructed a co-culture system of THP-1 cells and human T cells. It was found that knocking down Ripk2 in macrophages promoted the expression of IFN-γ, while overexpression of Ripk2 in macrophages inhibited the expression of IFN-γ in T cells (Fig. S3).

To further identify the cytokine involved in this process, we analyzed the highly variable genes along the pseudotime trajectory and found a marked increase in the cytokine Cxcl9 (Fig. 5J). Previous studies have shown that macrophage-derived Cxcl9 promotes the infiltration of CD8^+^ T cells into tumor tissues, thereby enhancing their ability to kill tumor cells [11]. ELISA detection showed that serum Cxcl9 levels were significantly elevated in both the Ripk2^*CKO*^ and GSK583 groups (Fig. 5K). FCM detection further revealed that the use of a Cxcl9 receptor inhibitor (AMG 487) could reverse the Ripk2 knockout-induced increase in CD3^+^ CD8^+^ cells and CD8^+^ GZMB^+^ cells (Fig. S4). These data indicate that targeting macrophage Ripk2 promotes cytotoxic CD8^+^ T cell recruitment *via* Cxcl9.

### Ripk2 deletion enhances T cell function through the Myb/Cxcl9 axis

**Fig. 6:**
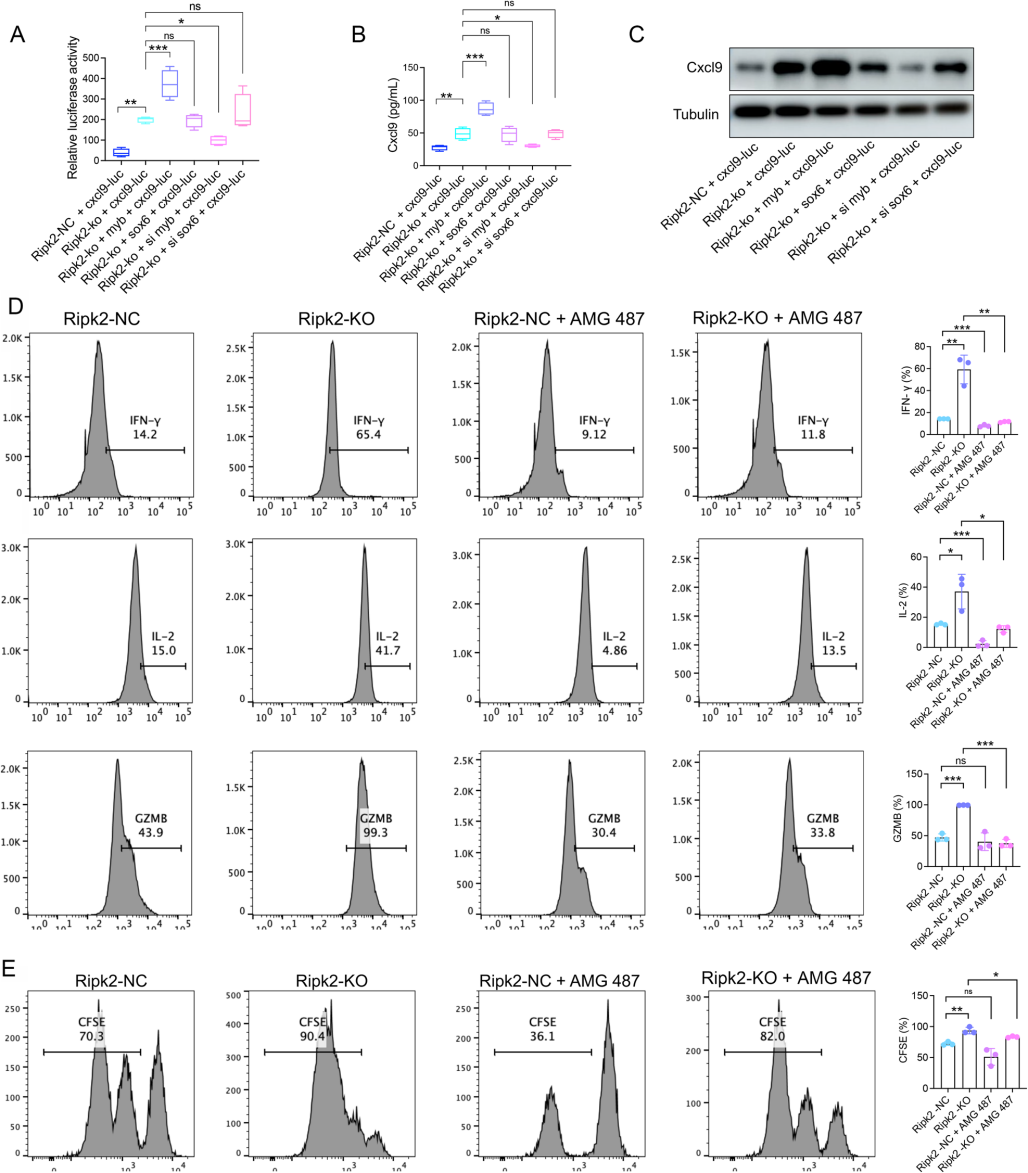
Deletion of Ripk2 promotes T cell proliferation and effector functions by activating Myb/Cxcl9. **A** We transfected pRL-TK, Cxcl9-luc, HA-sox6 and HA-Myb plasmids, as well as si Myb and si Sox6, into Ripk2-NC and Ripk2-KO cell lines, respectively. Dual-luciferase reporter gene assays were used to detect Cxcl9 promoter activity. *n* = 4. Data analysis was performed using ANOVA. **B** Cxcl9 secretion levels in cell culture supernatants were detected by ELISA. *n* = 4. Data analysis was performed using ANOVA. **C** Cxcl9 protein expression was detected by immunoblotting. *n* = 3. **D** T cell expression of IFN-γ, IL-2, and GZMB was detected by FCM. *n* = 3. Data analysis was performed using ANOVA. **E** T cell proliferation capacity was detected by FCM. *n* = 3. Data analysis was performed using one-way ANOVA. “ns” means no significant difference. *P < 0.05, **P < 0.01, ***P < 0.001.

Since inhibition of Ripk2 in macrophages can promote Cxcl9 secretion, we next investigated how Ripk2 affects Cxcl9 secretion. Specifically, we explored whether the deletion of Ripk2 influences Cxcl9 secretion through Myb or Sox6. We used website (http://jaspar.genereg.net/) to analyze the binding sequences of the transcription factors Myb and Sox6 with 2000 bp upstream of the Cxcl9 promoter (Table S2). We further used chromatin immunoprecipitation (ChIP) to verify that Myb, but not Sox6, binds to the Cxcl9 promoter (Fig. S5A). The dual luciferase reporter (DLR) experiment demonstrated that Myb activates the wild-type, but not a mutant, Cxcl9 promoter (Fig. S5B). Therefore, we hypothesized that the deletion of Ripk2 might increase Cxcl9 secretion by promoting Myb expression. DLR assays further demonstrated that Ripk2 deletion activated the Cxcl9 promoter, an effect enhanced by Myb overexpression but not by Sox6 (Fig. 6A). Knocking down Myb, but not Sox6, inhibited this activation (Fig. 6A), indicating that Ripk2 deletion promotes Cxcl9 promoter activity by activating Myb. ELISA and immunoblotting confirmed that Ripk2 deletion promotes Cxcl9 secretion and protein expression via Myb (Fig. 6B, C). Subsequently, in vitro experiments confirmed that the inhibition of Myb protein expression and Cxcl9 secretion by *Sc* were dependent on Ripk2 (Fig. S5C, D). These results collectively demonstrate that the deletion of Ripk2 affects Cxcl9 promoter activity through Myb, thereby promoting Cxcl9 expression and secretion.

To determine whether Cxcl9 induced by Ripk2 deletion affects the killing activity of T cells in vitro, we established a co-culture system of BMDMs and T cells. Ripk2 deficiency in BMDMs significantly augmented T cell activation, as demonstrated by markedly elevated secretion of IFN-γ, IL-2, and GZMB (Fig. 6D). This immunomodulatory effect was completely reversed by AMG 487 (Fig. 6D). These findings suggest that Ripk2 deletion activates T cell cytotoxic effects by inducing Cxcl9 secretion. To validate the killing ability of T cells induced by Cxcl9, we established a co-culture system of T cells and Hep53.4 cells. Cxcl9-derived peptide significantly increased the apoptosis level of Hep53.4 cells (Fig. S6A). In addition, Ripk2 deletion significantly promoted T cell proliferation, an effect that could also be inhibited by AMG 487 (Fig. 6E). These results confirm that Ripk2 deletion boosts CD8^+^ T cell cytotoxicity and proliferation by enhancing Cxcl9 secretion.

### Ripk2 enhances Adpgk-mediated glycolysis and promotes lactate production

**Fig. 7:**
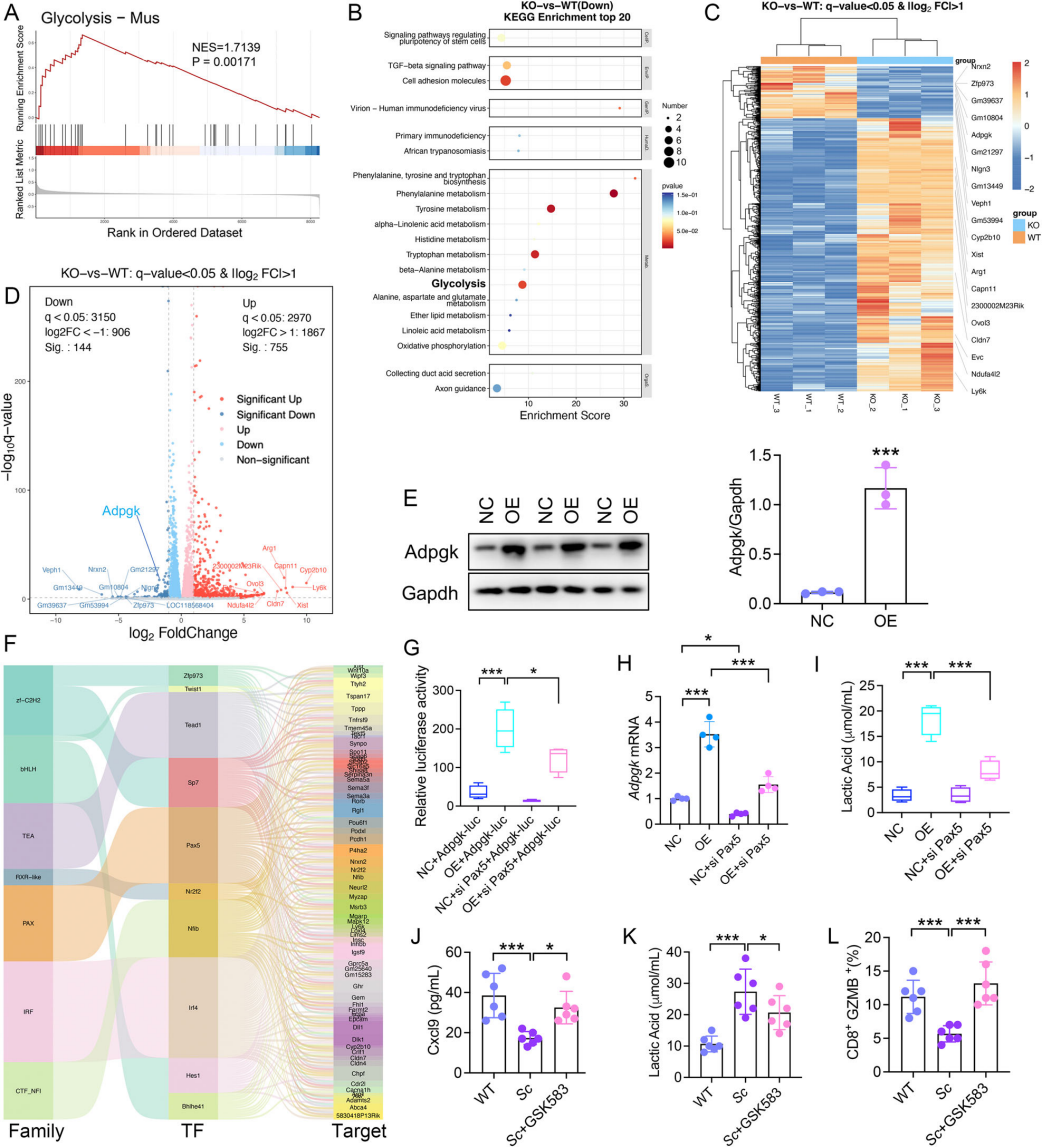
Ripk2 enhances Adpgk-mediated glycolysis and promotes lactate production. **A** GSEA analysis of scRNA-seq data. **B** KEGG analysis. **C** Differential gene heatmap of macrophages in Ripk2 knockout (KO) mice compared to WT mice. **D** Volcanic map of differentially expressed genes in macrophages of Ripk2 knockout (KO) mice compared to WT mice. **E** Immunoblotting was used to detect the effect of overexpression of Ripk2 on the expression level of Adpgk in vitro. *n* = 3. Data were mean ± SD and analyzed by t test. **F** The top 10 transcription factors affected by Ripk2^*CKO*^. **G** Dual-luciferase reporter gene assays were used to detect Adpgk promoter activity. *n* = 4. Data analysis was performed using ANOVA. **H** The mRNA level of *Adpgk* was analyzed by qRT-PCR. *n* = 4. Data were mean ± SD and analyzed by one-way ANOVA. **I** Lactate concentration in cell culture medium. *n* = 4. Data were mean ± SD and analyzed by ANOVA. **J** Cxcl9 secretion levels in mouse serum were detected by ELISA. *n* = 6. Data analysis was performed using ANOVA. **K** Lactate concentration in mouse serum. *n* = 6. Data were mean ± SD and analyzed by ANOVA. **L** FCM analysis of the proportions of CD8^+^ GZMB^+^ cells. *n* = 6. Data were mean ± SD and analyzed by ANOVA. *P < 0.05, ***P < 0.001.

Given the metabolic phenotype of M1_M macrophages, we investigated Ripk2’s role in macrophage metabolism. GSEA suggested a positive correlation between Ripk2 expression and glycolysis in mouse HCC macrophages (Fig. 7A). This finding contradicts our previous analysis which showed a negative correlation between Ripk2 expression and cellular metabolic signals. This discrepancy may be due to the fact that the total cell population represents a broader range of cell types, while the relationship between Ripk2 and metabolism in macrophages is our focus. Therefore, we further analyzed the relationship between Ripk2 and glycolysis in macrophages. We isolated macrophages from orthotopic HCC tissues of WT and Ripk2^*CKO*^ mice for RNA-seq. KEGG enrichment analysis demonstrated that knocking out Ripk2 inhibited glycolysis in macrophages (Fig. 7B). Next, we analyzed extracellular acidification rate (ECAR). Compared with macrophages in the WT group, macrophages in the Ripk2^*CKO*^ group exhibited lower glycolytic flux, glycolytic capacity, and glycolytic reserve (Fig. S6B), indicating that Ripk2 promotes glycolysis.

To evaluate the relationship between Ripk2 and glycolysis, we found that the expression of the glycolytic enzyme Adpgk was significantly decreased in Ripk2^*CKO*^ mouse macrophages by RNA-seq (Fig. 7C, D). Western blotting confirmed that overexpression of Ripk2 promotes Adpgk expression in vitro (Fig. 7E). We also conducted a comparative analysis of transcription factors affected by Ripk2^*CKO*^ and screened the top 10 transcription factors (Zfp973, Twist1, Tead1, Sp7, Pax5, Nr2f2, Nfib, Irf4, Hes1, and Bhlhe41) with the largest |log_2_FC| values (Fig. 7F). Among the seven transcription factors (Sp7, Pax5, Nr2f2, Nfib, Irf4, Hes1 and Bhlhe41) of Adpgk, the expression of Pax5 was significantly decreased. Therefore, we hypothesized that Ripk2^*CKO*^ disrupts Adpgk expression by inhibiting Pax5 expression. To test this hypothesis, we constructed an Adpgk promoter reporter gene. In Ripk2-overexpressing macrophages, we observed significant activation of the Adpgk promoter (Fig. 7G). This transcriptional upregulation was completely abolished by siPax5 (Fig. 7G), suggesting a Ripk2-Pax5 regulatory axis controls Adpgk expression through promoter interaction. qRT-PCR indicated that siPax5 could inhibit the mRNA level of *Adpgk* in BMDMs overexpressing Ripk2 (Fig. 7H). Furthermore, overexpression of Ripk2 promoted the production of glycolysis product lactate, which was inhibited by siPax5 (Fig. 7I), indicating that Ripk2 promotes lactate production through the Pax5/Adpgk signaling pathway. Lactate can promote CD8⁺ T cell dysfunction [12, 13]. Peritumoral injection of *Sc* decreased serum Cxcl9 levels (Fig. 7J), increased serum lactate (Fig. 7K), and reduced GZMB⁺ CD8⁺ T cell infiltration in tumors (Fig. 7L). These effects were fully reversed by GSK583 treatment (Fig. 7J–L), indicating Ripk2 mediates *Sc*-induced immunosuppression through chemokine regulation, metabolic reprogramming, and cytotoxic T cell inhibition. In addition, we also found that peritumoral injection of *Sc* depended on macrophages to inhibit the secretion level of Cxcl9, but not on CD8^+^ T cells (Fig. S6C). These results suggest that *Sc* activates Ripk2 signaling in macrophages, inhibits their secretion of Cxcl9, and promotes lactate production, leading to CD8^+^ T cell dysfunction.

### Combining GSK583 with a PD-1/PD-L1 inhibitor reduces immunotherapy-induced liver injury

**Fig. 8:**
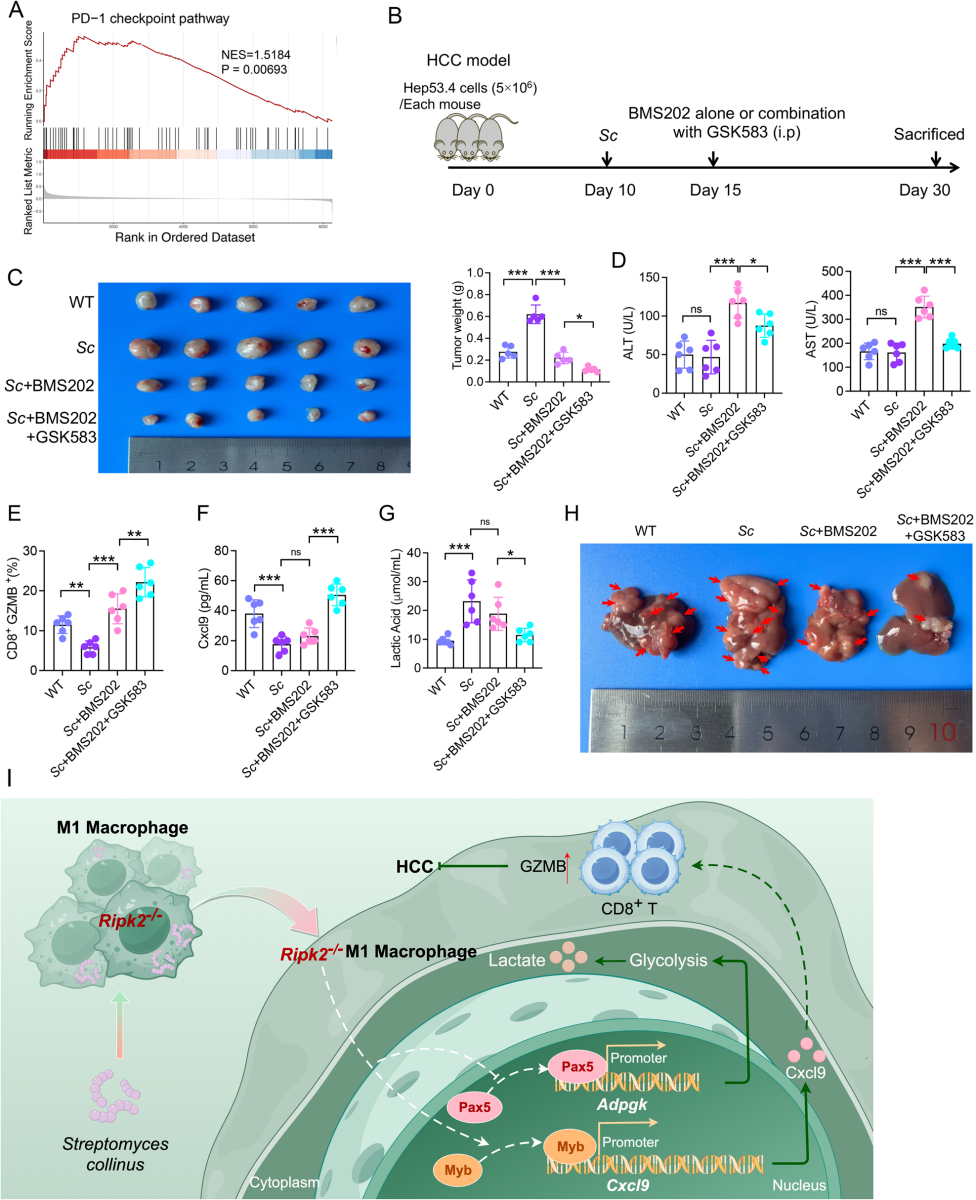
GSK583 combined with PD-1 inhibitors reduces liver injury. **A** GSEA analysis of scRNA-seq data. **B** Schematic diagram showing the use of BMS202 alone or in combination with GSK583 for the treatment of *Sc* induced HCC. **C** The effect of BMS202 alone or in combination on *Sc* induced HCC growth. *n* = 6. Data were mean ± SD and analyzed by one-way ANOVA. **D** AST and ALT levels were measured. *n* = 6. One-way ANOVA were used for statistical analysis. **E** FCM analysis of the proportions of CD8^+^ GZMB^+^ cells. *n* = 6. Data were mean ± SD and analyzed by one-way ANOVA. **F** Cxcl9 secretion levels in mouse serum were detected by ELISA. *n* = 6. Data analysis was performed using ANOVA. **G** Lactate concentration in mouse serum. n = 6. Data were mean ± SD and analyzed by ANOVA. **H** Observation of tumor nodules of orthotopic HCC infected with *Sc* after treatment with BMS202 alone or combined with GSK583. *P < 0.05, **P < 0.01, ***P < 0.001. **I** Working mode diagram. Inhibition or deletion of Ripk2 affects the intra-macrophage microbiome, upregulating Myb expression and enhancing CD8^+^ T cell activity. Additionally, Ripk2 promotes lactate production *via* Pax5/Adpgk, and combining a Ripk2 inhibitor with a PD-1 inhibitor improves immunotherapeutic efficacy for liver injury. This image was drawn by Figdraw.

Ripk2 modulates the inflammatory response through signaling initiated by the microbiota-sensing PRR molecules Nod1/2. Previous studies have shown that PRRs are activated during liver injury induced by immunotherapy with immune checkpoint inhibitors [14]. Furthermore, targeting Ripk2 can enhance the efficacy of anti-PD-1 treatment [15]. Our GSEA analysis revealed a positive correlation between Ripk2 expression and the PD-1 checkpoint pathway (Fig. 8A). During acute liver injury, high expression of PD-1 and PD-L1 in liver macrophages is associated with impaired antimicrobial function [16]. Therefore, we hypothesized that the combination of GSK583 and a PD-1/PD-L1 inhibitor can prevent liver injury caused by PD-1 inhibitor monotherapy in *Sc*-induced HCC, thereby restoring the functional activity of CD8^+^ T cells and enhancing immunotherapy efficacy. We treated subcutaneous HCC tumors with PD-1/PD-L1 inhibitor (BMS202) (Fig. 8B). Combination therapy with GSK583 significantly inhibited *Sc*-induced HCC growth compared to BMS202 monotherapy (Fig. 8C). Serological analysis showed that intratumoral injection of *Sc* did not cause liver injury, while BMS202 monotherapy did induce liver injury, which was reversed by combination therapy with GSK583 (Fig. 8D). FCM analysis found that the number of CD8^+^ GZMB^+^ T cells was significantly increased in the BMS202 combined with GSK583 group compared to the BMS202 monotherapy group (Fig. 8E). ELISA analysis indicated that BMS202 monotherapy had no effect on serum Cxcl9 levels. However, the serum Cxcl9 concentration was significantly higher in the BMS202 combined with GSK583 group compared to the BMS202 monotherapy (Fig. 8F). Additionally, BMS202 monotherapy had no effect on serum lactate levels; however, combination therapy with GSK583 inhibited lactate production (Fig. 8G). Furthermore, the orthotopic HCC model was used to verify that BMS202 combined with GSK583 significantly inhibited the growth of HCC (Fig. 8H). These data show that GSK583 combination therapy reduces liver injury and improves immunotherapy outcomes.

## Discussion

The presence of microbiota in HCC has been confirmed. However, the oncogenic or tumor-suppressive effects of intratumoral microbiota largely hinge on their modulation of the immune system. On one hand, microorganisms can elicit chronic inflammation and facilitate tumor progression; on the other hand, they can activate immune responses that eliminate tumor cells [17]. The overexpression of *Escherichia coli*-adapted TLR4 in HCC contributes to the formation of an immunosuppressive microenvironment [18]. HCC patients with a relatively higher abundance of *Pseudomonas* exhibit a better prognosis. Upon recognizing pathogen-associated molecular pattern proteins (PAMPs), Nod1/2 interacts with Ripk2 to facilitate the secretion of pro-inflammatory cytokines mediated by NF-κB and downstream signaling cascades, resulting in chronic inflammatory infiltration and mediating malignant proliferation, aggressive metastasis, immune escape, and drug resistance of tumors [19, 20]. This study found that the Ripk2 inhibitor GSK583 increased the abundance of *Sc* in macrophages. Moreover, Ripk2 positively correlates with PD-L1 expression and the PD-1 checkpoint pathway in macrophages. Research has shown that Ripk2 reduces the sensitivity of gliomas to temozolomide (TMZ) by inducing the expression of O-6-methylguanine-DNA methyltransferase (MGMT) and activating the NF-κB signaling pathway [21]. However, studies have also indicated that Ripk2 knockout can promote the infiltration of myeloid-derived suppressor cells (MDSCs) in bladder cancer tissues by enhancing the secretion of granulocyte colony-stimulating factor (GCSF), suggesting that Ripk2 can also inhibit tumor progression by regulating the TME [22].

Ripk2 is a crucial driver of immune evasion from cytotoxic T-cell killing. By facilitating NBR1-mediated autophagolysosomal degradation, Ripk2 suppresses MHC-I expression in pancreatic ductal adenocarcinoma (PDAC) cells, thereby promoting immune evasion from cytotoxic T-cell-mediated killing [15]. Upon effective activation by the bacterial component peptidoglycan (PDG), Ripk2 induces PD-L1 expression, resulting in immune evasion in oral cancer cells [23]. Previous studies have shown an important relationship between Ripk2 and macrophage M1 polarization [24]. Our research confirms that the knockout or inhibition of Ripk2 in macrophages can promote the transition of classical M1 macrophages towards a metabolic phenotype. This transition modulates the activity and proliferation of CD8^+^ T cells by influencing the secretion of cytokines and chemokines, particularly Cxcl9 [25]. Additionally, we have discovered that Ripk2 affects glycolytic metabolism in macrophages through the Pax5/Adpgk signaling pathway. Notably, the knockout or inhibition of Ripk2 not only enhances Cxcl9 secretion but also reduces the production of lactate, a glycolytic metabolite of macrophages. This is conducive to improving the immune microenvironment and enhancing the anti-tumor efficacy of CD8^+^ T cells. Our research confirmed that *Sc* inhibits CD8^+^T activity and function through Ripk2/Myb/Cxcl9 signaling. However, previous studies have found that *Streptomyces* could promote anti-tumor immune responses by recruiting CD8^+^T cells in PDAC [26]. It might be due to the different effects of the same genus on the immune microenvironment in different tumors [27].

The application of PD-1/PD-L1 inhibitors in tumor therapy has yielded promising results. Nonetheless, excessive immune responses can also elicit immune-related adverse events (irAEs), among which immune-related liver injury is relatively prevalent and exhibits the highest incidence rate [28]. This research confirms that co-administration of GSK583 with a PD-1/PD-L1 inhibitor reduces the hepatotoxicity observed with PD-1/PD-L1 inhibitor monotherapy, leading to an enhanced immunotherapeutic outcome. Previous studies have also demonstrated that inhibiting the Nod1/Ripk2 signaling pathway can ameliorate liver damage in cholestatic rats [29]. Consequently, Ripk2 inhibitors play a significant role in suppressing the progression of liver injury.

## Conclusions

Our findings demonstrate that Ripk2 inhibition enhances both the efficacy of PD-1/PD-L1 blockade and mitigates its associated liver injury. Furthermore, we identify Ripk2 as a key regulator of *Sc* abundance in macrophages. Therefore, targeting Ripk2 presents a promising strategy to improve immunotherapy outcomes in HCC.

## Supplementary information

Supplementary Figures and Tables

Full and uncropped western blots

aj-checklist

## Data Availability

Most of the data and supplementary data have been provided in the article. The raw sequence data reported in this paper have been deposited in the Genome Sequence Archive (Genomics, Proteomics & Bioinformatics 2021) in National Genomics Data Center (Nucleic Acids Res 2022), China National Center for Bioinformation/Beijing Institute of Genomics, Chinese Academy of Sciences (GSA: CRA022377) that are publicly accessible at https://ngdc.cncb.ac.cn/gsa. Other data can be provided upon reasonable request.
