# Supplementary material for: Ripk2 promotes CD8+ T cell inactivation and hepatocellular carcinoma progression through Myb/Cxcl9 and Pax5/Adpgk signaling pathways
Source: Cell Death Dis. 2026 May 29;17(1):663. doi: 10.1038/s41419-026-08849-0 (PMC13408793; doi:10.1038/s41419-026-08849-0)
Supplement: Supplementary file 1 — Supplementary Figures and Tables [file 41419_2026_8849_MOESM1_ESM.pdf]

Supplementary information for

Ripk2 promotes CD8$^+$ T cell inactivation and hepatocellular carcinoma progression through Myb/Cxcl9 and Pax5/Adpgk signaling pathways

Table of contents

Supplementary Figures………………………………………………………………………2

Supplementary Tables……………………..……………………………………………………7

Supplementary Figures

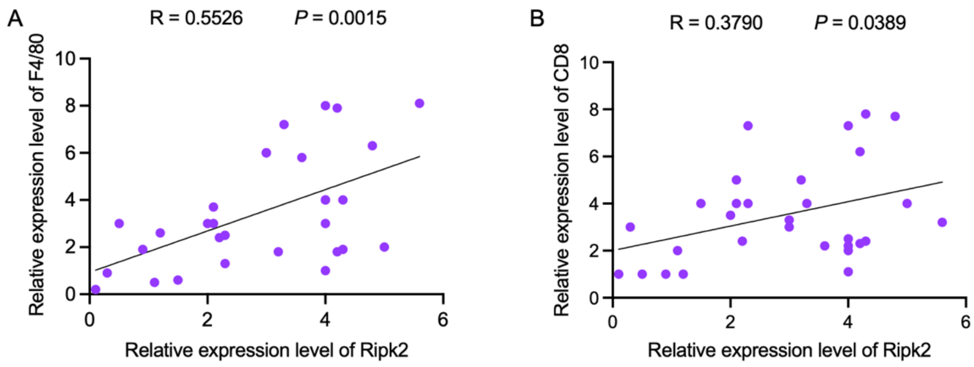

Fig S1 Correlation between Ripk2, F4/80 and CD8 in human HCC. (A) and (B) Pearson correlation analysis of the correlation between Ripk2, F4/80 and CD8 in human HCC. n=30.

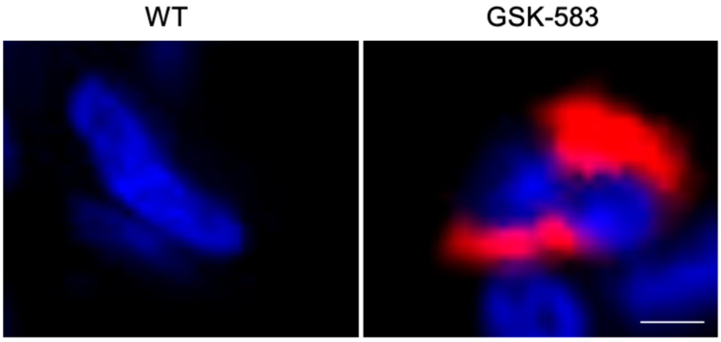

Fig S2 FISH detection of phagocytosis of *Sc* by macrophages sorted from tumor tissues.

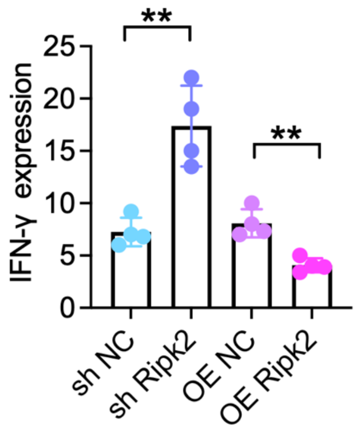

Fig S3 FCM analysis of the level of IFN-γ expression in T cells. n=4. Data were mean ± SD and analyzed by ANOVA. ***P* < 0.01.

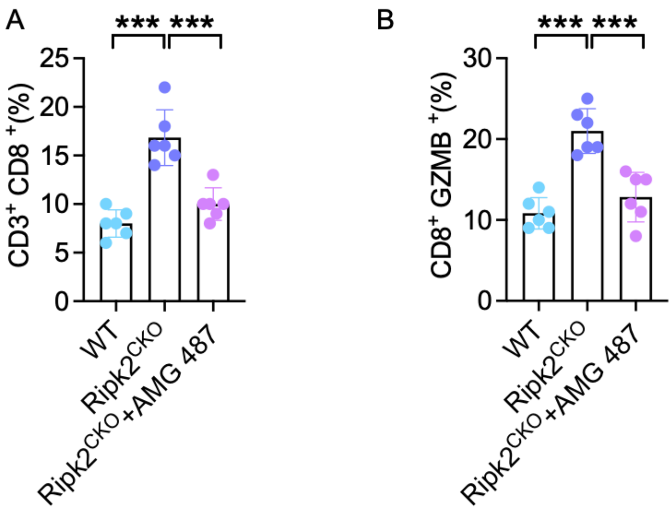

Fig S4 FCM analysis of the impact of AMG 487 on the proportions of $CD3^{+}CD8^{+}$ cells and $CD8^{+}GZMB^{+}$ cells. n=6. Data were mean ± SD and analyzed by ANOVA. ***$P < 0.001$.

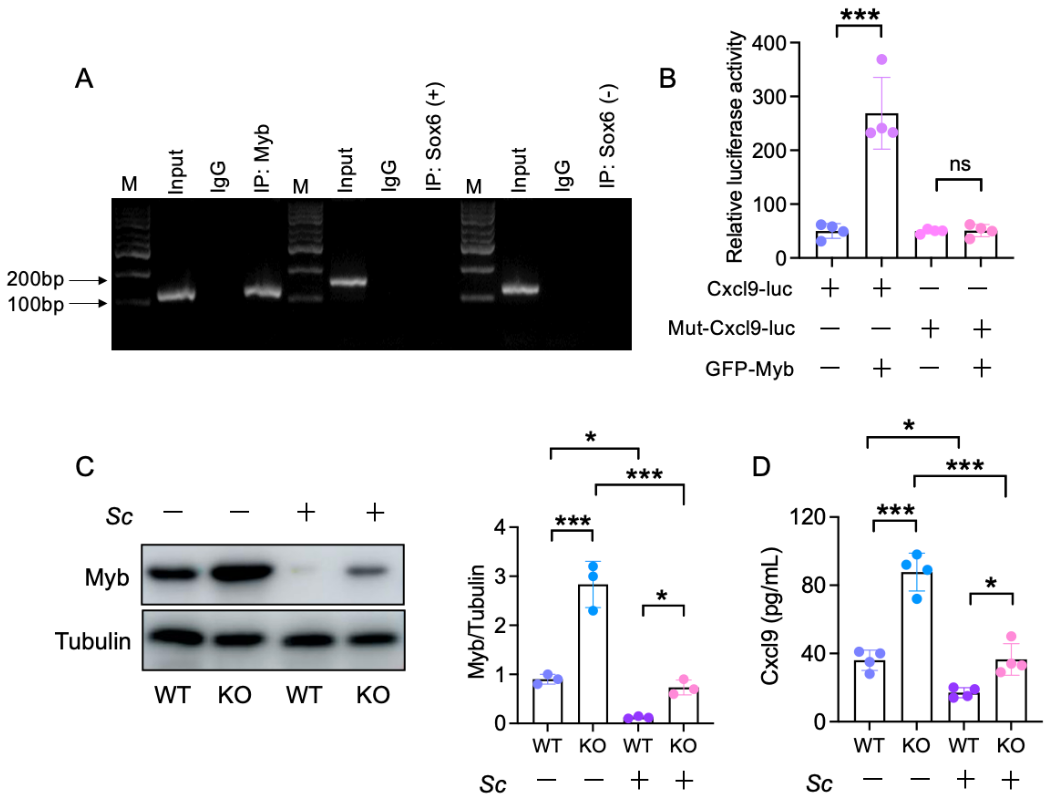

Fig S5 Myb activates the promoter of Cxcl9. (A) The ChIP experiment indicated that Myb bound to the promoter of Cxcl9. (B) The DLR assay was used to analyze the activity of the Cxcl9 promoter in BMDMs transfected with or without the GFP-Myb. n=4. Data were mean ± SD and analyzed by one-way ANOVA. ***$P < 0.001$. "ns" indicates no significant difference. (C) Western blotting was used to detect the effect of *Sc* infection on the expression level of Myb protein in BMDMs with and without Ripk2 gene knockout. Data were mean ± SD and analyzed by one-way ANOVA. *$P < 0.05$, ***$P < 0.001$. (D) ELISA was used to analyze the secretion level of Cxcl9 in the cell supernatant. Data were mean ± SD and analyzed by one-way ANOVA. *$P < 0.05$, ***$P < 0.001$.

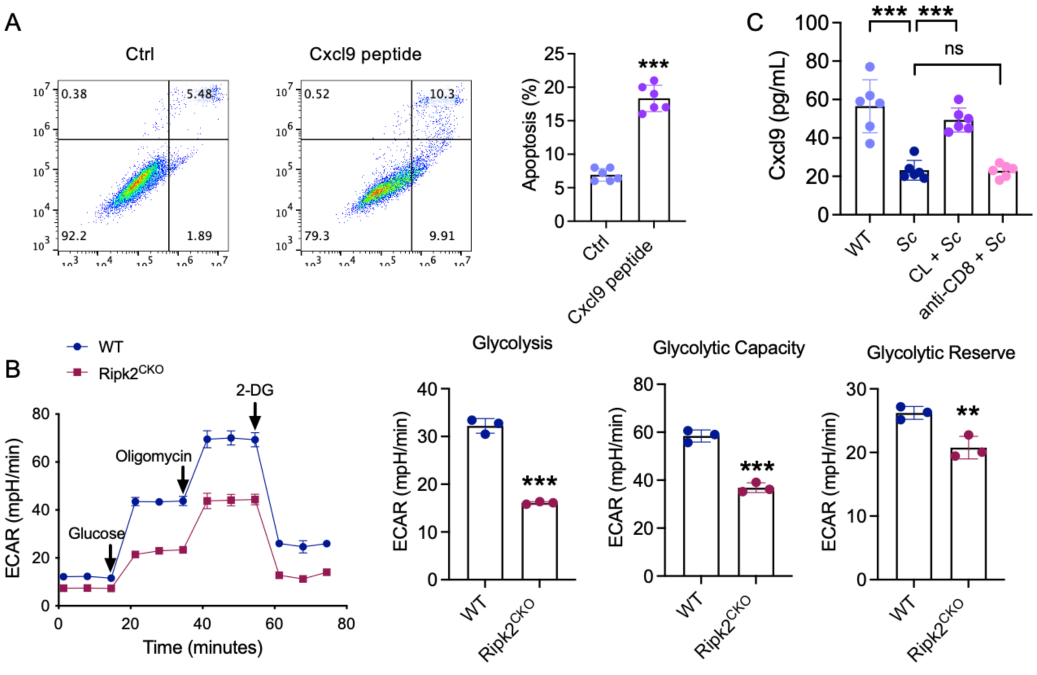

Fig S6 Ripk2 promotes glycolysis in macrophages. (A) Analysis of apoptosis level of Hep53.4 cells. n=6. Data were mean ± SD and analyzed by t test. ***$P < 0.001$. (B) Macrophages were used for ECAR detection of glycolytic flux, glycolytic capacity and glycolytic reserve. n=3. Data was analyzed using t tests. **$P < 0.01$; ***$P < 0.001$. (C) Before establishing subcutaneous tumors, macrophages and CD8 cells in mice were cleared using CL and anti-CD8 respectively. Cxcl9 secretion levels in mouse serum were detected by ELISA. n=6. Data analysis was performed using one-way ANOVA. ***$P < 0.001$.

Supplementary Tables

Table S1. Enrichment analysis of signal pathways.

Description	GeneRatio	BgRatio	pvalue	p.adjust
NF-kappa B signaling pathway - Mus musculus (house mouse)	20/334	107/9772	4.29E-10	3.79E-08
Chemokine signaling pathway - Mus musculus (house mouse)	25/334	193/9772	8.57E-09	4.54E-07
TNF signaling pathway - Mus musculus (house mouse)	18/334	118/9772	8.97E-08	3.39E-06
T cell receptor signaling pathway - Mus musculus (house mouse)	16/334	122/9772	3.71E-06	7.02E-05
Toll-like receptor signaling pathway - Mus musculus (house mouse)	12/334	104/9772	0.00021383	0.00217938
IL-17 signaling pathway - Mus musculus (house mouse)	11/334	93/9772	0.00031377	0.00307957
p53 signaling pathway - Mus musculus (house mouse)	9/334	75/9772	0.00098334	0.00840596
NOD-like receptor signaling pathway - Mus musculus (house mouse)	17/334	216/9772	0.00116918	0.00938887
Hepatitis B - Mus musculus (house mouse)	14/334	164/9772	0.001456	0.01134823

Table S2 The binding sequences of the transcription factors Myb and Sox6

Name	Score	Relative score	Start	End	Strand	Predicted sequence
MA0100.2.Myb	12.812772	0.9588327	1661	1670	-	CAAACTGCCT
MA0515.1.Sox6	11.766686	0.9387826	1197	1206	+	GCTTTGTTCC
MA0515.1.Sox6	11.319688	0.9302634	1141	1150	+	CTTTTGTTTT
MA0515.1.Sox6	10.516774	0.914961	812	821	-	GTATTGTTCT
