# Supplementary figures and images for: Ripk2 promotes CD8+ T cell inactivation and hepatocellular carcinoma progression through Myb/Cxcl9 and Pax5/Adpgk signaling pathways
Source: Cell Death Dis. 2026 May 29;17(1):663. doi: 10.1038/s41419-026-08849-0 (PMC13408793; doi:10.1038/s41419-026-08849-0)

Fig 2f

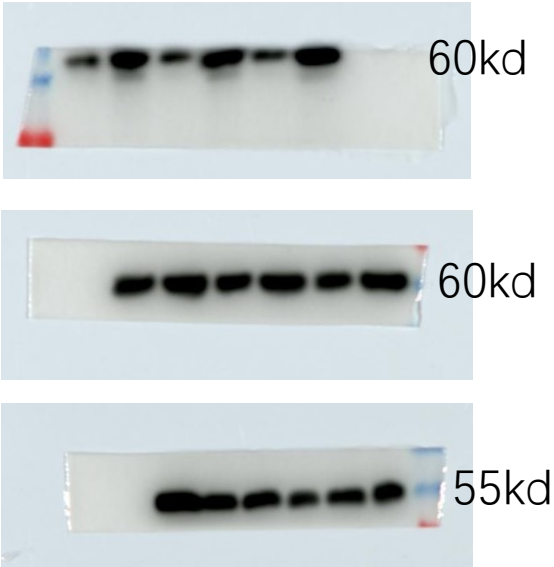

Fig 3e

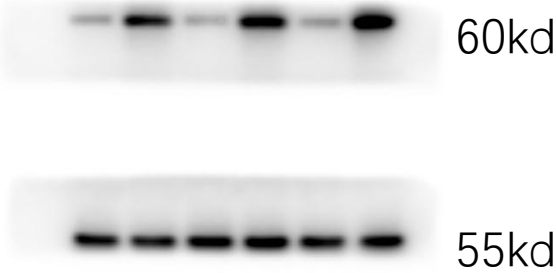

Fig S5C

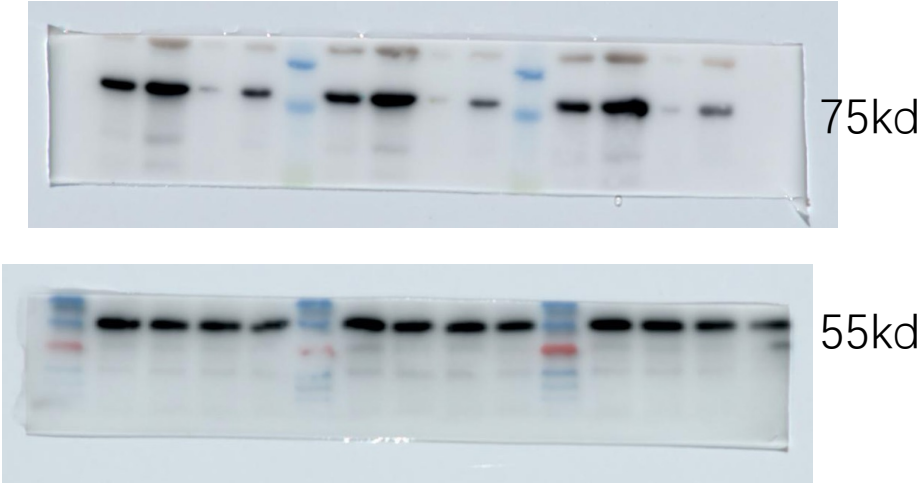

Fig 6c

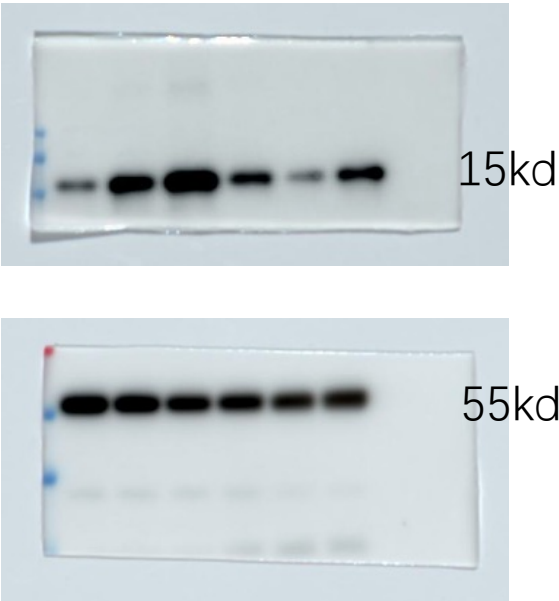

Fig 7e

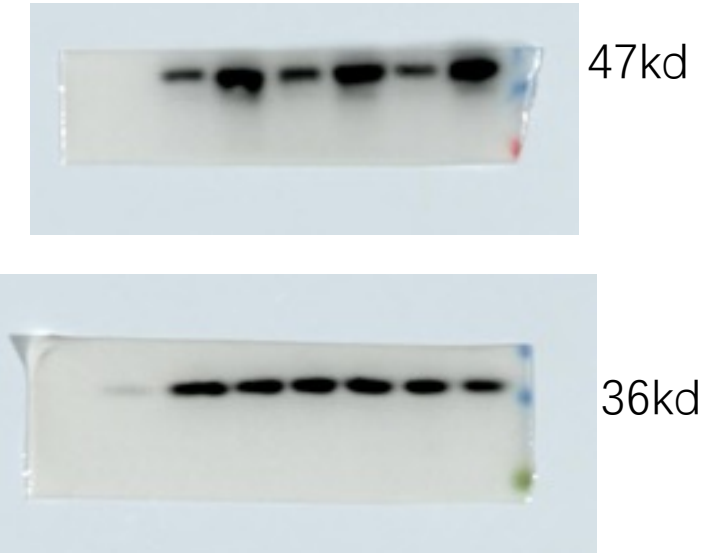

Supplement: Supplementary file 2 — Full and uncropped western blots [file 41419_2026_8849_MOESM2_ESM.pdf]
